# ﻿A new species of *Monomorium* Mayr, 1855 (Hymenoptera, Formicidae) with a brachypterous queen from southern Japan, and phylogeny of Japanese *Monomorium* species with diverse reproductive strategies

**DOI:** 10.3897/zookeys.1250.150776

**Published:** 2025-08-26

**Authors:** Naoto Idogawa, Shigeto Dobata, Yasukazu Okada, Seiki Yamane

**Affiliations:** 1 Department of Biological Science, School of Science, Nagoya University, Furo-cho, Chikusa-ku, Nagoya 464-8602, Japan Nagoya University Nagoya Japan; 2 Department of General Systems Studies, Graduate School of Arts & Sciences, University of Tokyo, Komaba 3-8-1, Meguro-ku, Tokyo 153-8902, Japan University of Tokyo Tokyo Japan; 3 Haruyama-cho, Kagoshima, 899-2704, Japan Unaffiliated Kagoshima Japan

**Keywords:** *COI* gene, colony foundation, dispersal strategy, identification key, mitochondrial genome, *Monomorium intrudens*, nest size, pest species, protein-coding genes, Ryukyu Islands, sister species, social structure, tramp species, wing morphology

## Abstract

The genus *Monomorium* is an important phylogenetic group, notable for its taxonomic complexity and the presence of several well-known tramp species. In this study, we present a description of *M. brachypterum***sp. nov.** from southern Japan along with an updated key to the Japanese species of the genus based on the worker caste. Molecular phylogenetic analysis using 13 protein-coding genes of the mitochondrial genome indicated that this new species is most closely related to *M. intrudens*. Within their overlapping distributions, analyses based on partial sequences of the mitochondrial *COI* gene showed that the two species are phylogenetically distinct. The queens of *M. brachypterum* are distinguished from those of *M. intrudens* by degenerated wings, suggesting contrasting dispersal strategies between the two sister species. In addition, a phylogenetic analysis performed for the Japanese *Monomorium* species provides support for the repeated evolution of diverse reproductive systems within this genus. The present study highlights a wide variety of evolutionary trends in the nest-level reproductive system, even among closely related species.

## ﻿Introduction

*Monomorium* Mayr, 1855 is a large and taxonomically problematic genus in the hyperdiverse ant subfamily Myrmicinae, with 297 species described (Bolton 2024) and potentially hundreds of species remaining undescribed (Sharaf et al. 2021; Andersen et al. 2022). The genus has a long and complex taxonomic history, with major revisions in different biogeographic regions: Afrotropical species were comprehensively reviewed by Bolton (1987), Malagasy species by Heterick (2006), Australian species by Heterick (2001, 2003, 2009), and Nearctic species by DuBois (1986) and Mackay and Mackay (2002), and Arabian Peninsula species by Sharaf et al. (2021). Based on the comprehensive molecular phylogenetics (Ward et al. 2015), some groups formerly included in *Monomorium* have been resurrected or newly established as independent genera in the last decade (Fisher and Bolton 2016; Sparks et al. 2019). As a result, some widespread tramp species such as *Trichomyrmex destructor* (Jerdon, 1851) (Wetterer 2009), *Syllophopsis sechellensis* (Emery, 1894) (Wetterer and Sharaf 2017) and *Erromyrma latinodis* (Mayr, 1872) (Sharaf et al. 2018) were removed from *Monomorium*. Nevertheless, this genus still includes notorious pest species such as *M. pharaonis* (Linnaeus, 1758) (Wetterer 2010a) and *M. floricola* (Jerdon, 1851) (Wetterer 2010b) and remains an important group in the context of pest management and invasion biology. In addition to cosmopolitan tramp species, the genus is ecologically highly diverse, including generalist scavengers as well as species that harvest seeds, exhibit lestobiotic behavior, or engage in social parasitism. *Monomorium* species occupy a wide range of environments, from the arid Sahara to tropical rainforests (Ettershank 1996).

The center of diversity of *Monomorium* lies in the Afrotropical region, with relatively few species found in East Asia. In Japan, only six species − *M. chinense* Santschi, 1925, *M. floricola*, *M. hiten* Terayama, 1996, *M. intrudens* Smith, 1874, *M. pharaonis* and *M. triviale* Wheeler, 1906 − have been reported from natural habitats. It has been suggested that *M. chinense*, *M. floricola* and *M. pharaonis* may have been introduced through human activity (Terayama et al. 2014). In addition, *M. salomonis* (Linnaeus, 1758), *M. sahlbergi* Emery, 1898 and *M.* sp. related to *M. salomonis* have recently been detected in Japanese port areas and imported cargo (Terayama et al. 2022), suggesting potential invasion risks associated with this genus.

**Figure 1.**
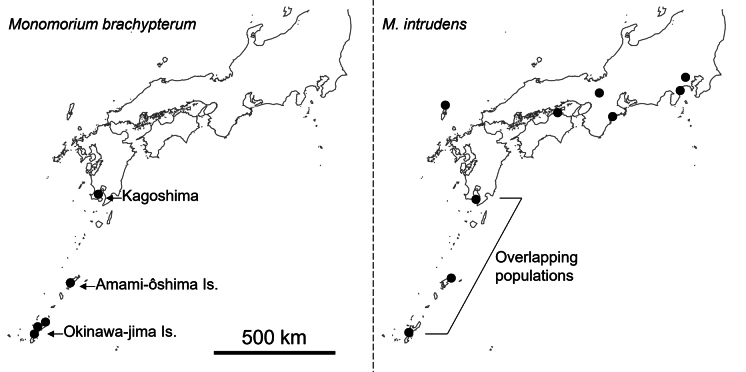
Sampling sites for *Monomorium brachypterum* and *M. intrudens* in central and southern Japan. Arrows indicate all known populations of *M. brachypterum*, which occur exclusively in areas where *M. intrudens* is also present.

During a continuous collection of *Monomorium intrudens* across Japan since 2017 (Suppl. material 1: table S1), we noticed that some samples from southern Japan exhibited peculiar phenotypes (Fig. 1). Based on morphological and molecular evidence, these samples were later considered to belong to a different species, new to science, and to be the sister species of *M. intrudens*. Here we provide descriptions of all the adult sexes/castes of a new species, *M. brachypterum* sp. nov., with information on its social structure. Furthermore, we also show the phylogenetic relationship of East Asian members of *Monomorium*, together with five newly assembled complete mitochondrial genomes, for future taxonomic studies.

## ﻿Materials and methods

### ﻿Sampling

To investigate the social structure of *M. brachypterum* sp. nov. and *M. intrudens*, we counted the number of queens and workers after collection. These samples from multiple sampling sites were also subjected to phylogenetic analysis based on mitochondrial cytochrome oxidase I (*COI*) gene sequence and morphological observations. To determine their phylogenetic position among congeners, we collected colonies of additional Japanese *Monomorium* species (Table 1) from their natural habitats for complete mitochondrial genome sequencing. For all the collected species, colonies were fixed in 99.5% ethanol and frozen at –80 °C until DNA extraction or morphological observations.

**Table 1.** Sequenced materials and their sampling information.

GenBank_ID	Species	Sequence	Sampling_date	Location	GPS code (°)
LC859928	*Monomorium chinense*	Mitogenome, *COI*	2017-09-25	Japan:Osaka,Sakai-shi	34.5459°N, 135.5062°E
LC859929	*Monomorium floricola*	Mitogenome	2023-07-10	Japan:Okinawa,Nakijin-son	26.6946°N, 127.9281°E
LC859930	*Monomorium hiten*	Mitogenome	sampled from artificially reared colony	Japan:Okinawa, Yonaguni	NA
LC859931	*Monomorium intrudens*	Mitogenome, *COI*	2019-05-27	Japan:Mie,Minami-ise-cho	34.2812°N, 136.5966°E
LC859932	*Monomorium brachypterum*	Mitogenome, *COI*	2023-03-19	Japan:Kagoshima,Setouchi-cho	28.2219°N, 129.3413°E
LC858795	*Monomorium brachypterum*	*COI*	2023-01-29	Japan:Okinawa,Uruma-shi	26.4068°N, 127.8175°E
LC858796	*Monomorium brachypterum*	*COI*	2023-07-10	Japan:Okinawa,Nakijin-son	26.6946°N, 127.9281°E
LC858797	*Monomorium brachypterum*	*COI*	2023-09-04	Japan:Okinawa,Kunigami-son	26.8329°N, 128.2888°E
LC858798	*Monomorium brachypterum*	*COI*	2023-03-29	Japan:Kagoshima,Uken-son	28.2464°N, 129.3277°E
LC858800	*Monomorium brachypterum*	*COI*	2018-07-07	Japan:Kagoshima,Uken-son	NA
LC858801	*Monomorium brachypterum*	*COI*	2018-07-07	Japan:Kagoshima,Uken-son	NA
LC858799	*Monomorium brachypterum*	*COI*	2023-03-19	Japan:Kagoshima,Yamato-son	28.3245°N, 129.3216°E
LC858802	*Monomorium brachypterum*	*COI*	2020-03-25	Japan:Kagoshima,Kagoshima-shi	31.4431°N, 130.4952°E
LC858803	*Monomorium intrudens*	*COI*	2023-03	Japan:Kagoshima,Ibusuki-shi	31.2391°N, 130.6336°E
LC858804	*Monomorium intrudens*	*COI*	2019-11-23	Japan:Okinawa,Onna-son	26.4648°N, 127.8301°E
LC858805	*Monomorium intrudens*	*COI*	2023-03-27	Japan:Kagoshima,Tatsugo-cho	28.4142°N, 129.6021°E
LC858806	*Monomorium intrudens*	*COI*	2023-08-19	Japan:Kanagawa,Manaduru-cho	35.1420°N, 139.1598°E
LC858807	*Monomorium intrudens*	*COI*	2023-06-21	Japan:Nagasaki,Tsushima-shi	34.6197°N, 129.3533°E
LC858808	*Monomorium intrudens*	*COI*	2018-12-22	Japan:Kagawa,Miki-cho	34.2713°N, 134.1224°E
LC858809	*Monomorium intrudens*	*COI*	2018-09-13	Japan:Kyoto,Kyoto-shi	35.0281°N, 135.7859°E
LC858810	*Monomorium intrudens*	*COI*	2023-05-09	Japan:Tokyo,Hachioji-shi	35.6211°N, 139.3872°E

### ﻿Phylogenetic analysis

To clarify the phylogenetic relationships of our new species and *M. intrudens* with other Japanese congeners, we sequenced the complete mitochondrial genomes of *Monomorium* species established in Japan, following Idogawa et al. (2021a) with a slight modification. Three species (*M. salomonis*, *M. sahlbergi*, and *M.* sp. related to *M. salomonis*), which are known only from port areas and are not considered established, were excluded from the analysis. We extracted genomic DNA from approximately 100 individuals using the DNeasy Blood and Tissue kit (Qiagen, Hilden, Germany). We sequenced the pooled DNA on the HiSeq X sequencer (Illumina, San Diego, CA) using a 150 paired-end sequencing strategy (approximately 5 Gb in size) at Macrogen Japan Corp. (Tokyo, Japan). After removing adapters with fastp ver. 0.12.4 (Chen et al. 2018), we performed *de novo* mitogenome assembly using NOVOPlasty ver. 3.6 (Dierckxsens et al. 2017) and GetOrganelle ver. 1.7.5.3 (Jin et al. 2020), with the *Monomorium pharaonis* mitogenome (NCBI Reference Sequence: NC_051486.1) as a seed. The sequence information was deposited in the DDBJ under accession number: LC859928 to LC859932 (Table 1).

We annotated the set of 13 protein-coding genes (PCGs), rRNAs, and tRNAs using MITOS2 (Bernt et al. 2013) and NCBI ORF-finder (Rombel et al. 2002), based on the assembled mitochondrial genomes, with average read coverage ranging from 26.4 to 5943.9. We inferred the phylogenetic relationships of the seven *Monomorium* species using the concatenated nucleotide sequences of all the PCGs (10,711 bp), with the red imported fire ant *Solenopsis invicta* (NC_014672.1) and the black imported fire ant *S. richteri* (NC_014677.1) as outgroups.

To ensure reproductive isolation between newly discovered species and *M. intrudens* in their overlapping distribution, phylogenetic analysis based on the mitochondrial *COI* gene was performed. A 658 bp barcode region of the *COI* sequence was determined by using PCR with a combination of primers, namely LCO1490 (GGTCAACAAATCATAAAGATATTGG) and HCO2198 (TAAACTTCAGGGTGACCAAAAAATCA). One randomly picked worker was sequenced from each of the eight nests of the new species and eight nests of *M. intrudens* (Table 1). Genomic DNA extraction was performed with the DNeasy Blood and Tissue kit (Qiagen, Hilden, Germany). PCR was done with Takara Ex Taq (Takara Bio, Otsu, Japan) and the thermal cycle with an initial denaturation at 94 °C for 1 min, 35 cycles of denaturation at 94 °C for 30 s, annealing at 50 °C for 30 s, extension at 72 °C for 60 s, and a final extension at 72 °C for 10 min. A total of 16 sequences were sequenced by Eurofins Genomics (Tokyo, Japan) and submitted to the DNA Data Bank of Japan (DDBJ) under accession numbers LC858795 to LC858810; see Fig. 3). The partial *COI* gene of the new species (DDBJ accession number LC859932) and *M. intrudens* (LC859931) were extracted from their complete mitochondrial genomes as additional sequences. As outgroups, *M. chinense* (DDBJ accession number LC859928) and *M. triviale* (LC605004) were included in the dataset.

In both phylogenetic analyses, sequence alignments were constructed using ClustalW (Thompson et al. 2003), as implemented in MEGA-X (Kumar et al. 2018). The best-fit models (GTR+I for *COI* and GTR+I+G for complete mitogenome) were determined by ModelTest-NG ver. 0.1.6 (Darriba et al. 2020). The maximum-likelihood tree was constructed using IQ-TREE ver. 2.3.6 (Minh et al. 2020) and the Bayesian inference tree using MrBayes ver. 3.2.7 (Ronquist et al. 2012).

### ﻿Morphological observations

For the morphological comparison of the newly described species and *M. intrudens*, we examined individuals from both sympatric and non-sympatric populations. For the newly described species, we analyzed 20 workers, 20 queens, and 28 males, all from sympatric populations (Fig. 1). For *M. intrudens*, we analyzed 15 workers, 10 queens, and 10 males from sympatric populations, and an additional 10 individuals of each caste from non-sympatric populations. To compare the body size of the two species, we measured the head width of the workers, alate queens and males. In the queens and the males, the area of the right fore- and hindwings was measured as well as other body parts mentioned below. We observed the individuals using the binocular microscope Leica S9i (Leica Microsystems, Wetzlar, Germany) and measured each body part using ImageJ software ver. 1.53e (Schneider et al. 2012). The raw measurement data are available in Suppl. material 2: table S2.

For the species description, some ethanol-preserved specimens were mounted. The pictures were taken using an OM SYSTEM OM-1 camera (OM Digital Solutions Corporation, Tokyo, Japan) equipped with a M.ZUIKO DIGITAL ED 75–300 mm F4.8–6.7 II lens (OM Digital Solutions Corporation), connected to either a Mitutoyo M Plan APO 5× / 0.14 (Mitutoyo Corporation, Kanagawa, Japan) or Olympus UM Plan Fl 10× / 0.30 microscope objective (Evident Corporation, Tokyo, Japan). The images were then processed using Zerene Stacker ver. 1.04. Terminology principally follows Bolton (1994) and Boudinot (2015) with slight modifications. Wing morphology, specifically the terminology, is based on Yoshimura and Fisher (2011). The measurements of the following body parts were performed with ImageJ ver. 1.53e (Schneider et al. 2012), as shown in Suppl. material 2: table S2.

**TL** total body length roughly measured from tip of head to tip of gaster;

**HW** maximum head width in full-face view, excluding eyes;

**HL** maximum head length in full-face view, measured from anterior clypeal margin to midpoint of a line drawn across posterior margin of head;

**EW** maximum width of eye;

**EL** maximum length of eye;

**SL** scape length excluding basal constriction and condylar bulb;

**PrW** maximum width of pronotum, measured in dorsal view;

**PtW** maximum width of petiole, measured in dorsal view;

**PptW** maximum width of postpetiole, measured in dorsal view;

**CI** cephalic index, HW/HL× 100;

**SI** scape index, SL/HW× 100.

Materials examined were deposited at the Institute of Tropical Agriculture, Kyushu University (**KUEC**, Fukuoka, Japan), Sk. Yamane Collection at the Kitakyushu Museum of Natural History and Human History (**SKYC**, Kitakyushu, Japan), and the F. Ito Collection at Kagawa University (Kagawa, Japan).

### ﻿Statistical analysis

The altitude of the sampling sites, the numbers of workers and queens in a nest and the head width of each caste were compared between *M. brachypterum* sp. nov. and *M. intrudens* using the Mann–Whitney U-test. We analyzed the allometric relationships between the head width and the square root of the wing area in alates across species using analysis of covariance (ANCOVA). Allometric data were natural log-transformed prior to the analysis. All statistical analyses were performed in R ver. 4.3.1 (R Core Team 2023).

## ﻿Results

### ﻿Phylogenetic analyses

**Figure 2.**
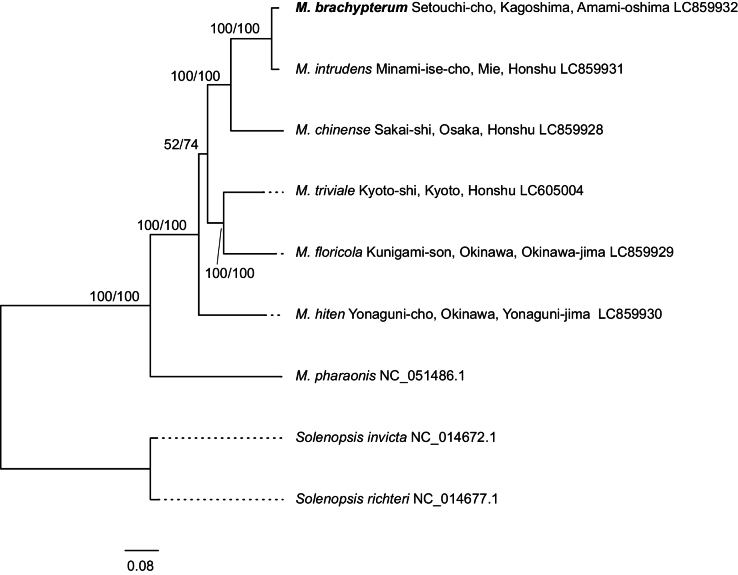
Maximum-likelihood (10000 bootstrap repeats) and Bayesian inference (100,000 generations) tree showing phylogenetic relationships among seven *Monomorium* species based on 13 protein-coding genes (PCGs) from complete mitochondrial genome sequences. Two *Solenopsis* species were used as the outgroup. The phylogenetic tree shown is based on the maximum-likelihood tree. The new species, *M. brachypterum*, is shown in bold. The numbers above the branches indicate maximum-likelihood bootstrap support (left) and Bayesian inference posterior probability (right). Original sequences and trees are available in Suppl. materials 6–8.

**Figure 3.**
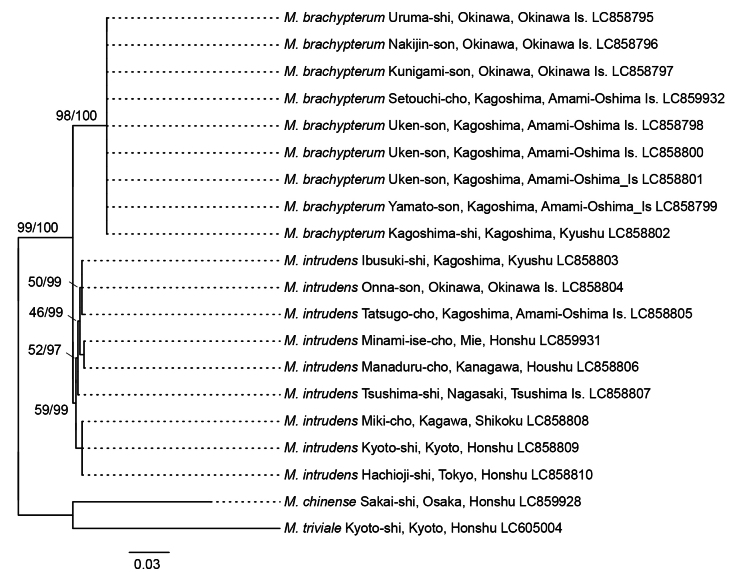
Maximum-likelihood (10000 bootstrap repeats) and Bayesian inference (100,000 generations) trees showing phylogenetic relationships between *Monomorium brachypterum* and *M. intrudens* based on 658 bp mitochondrial *COI* sequences. Two *Monomorium* species were used as the outgroups. The phylogenetic tree shown is based on the maximum-likelihood tree. The numbers above the branches indicate maximum-likelihood bootstrap support (left) and Bayesian inference posterior probability (right). Original sequences and trees are available in Suppl. materials 3–5.

The two partly sympatric species, *M. brachypterum* sp. nov. and *M. intrudens*, formed distinct clades with high support values in both the complete mitogenome and *COI* sequence analyses (Figs 2, 3). The lengths of the newly assembled mitogenomes of *Monomorium* species ranged from 16,034 bp in *M. intrudens* to 16,796 bp in *M. floricola*, with gene orders consistent with those previously reported for congeneric species (Idogawa et al. 2021a). The GC content of the entire genome ranged from 17% (*M. floricola*) to 21% (*M. hiten*). In these analyzed species, the new species was suggested to have diverged most recently from *M. intrudens*. The substitution rate of *COI* gene sequence between two species was 3.27% (50/1528 bp). Applying the calibration provided for Formicidae (Resende et al. 2010, 1.455% per site per million years), it is estimated that these two species diverged about 2.25 million years ago. Additionally, the thelytokous parthenogenetic species *M. triviale* is closely related to the sexual species *M. floricola* (Fig. 2).

### ﻿Nesting biology and social structure

**Figure 4.**
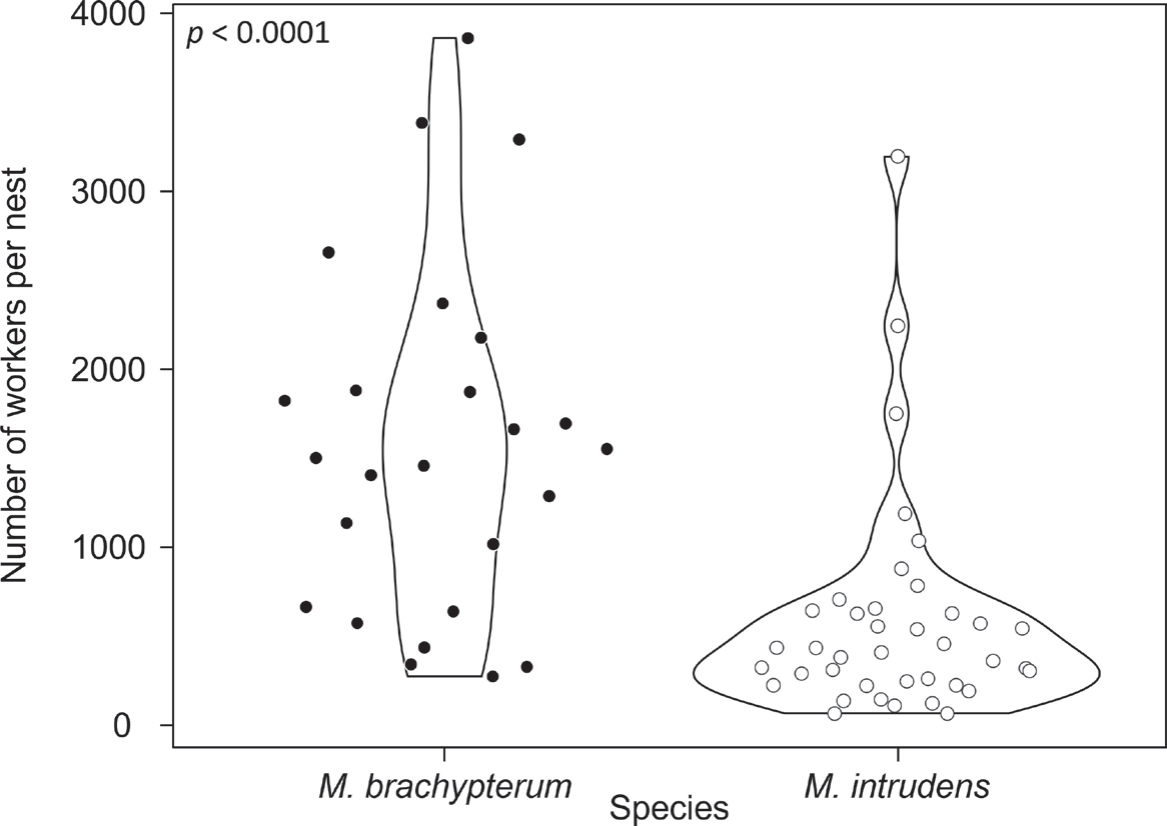
Nest size of *Monomorium brachypterum* (*N* = 25) and *M. intrudens* (*N* = 39).

In each habitat, both *M. intrudens* and *M. brachypterum* sp. nov. mainly nested in the dead stems of plants such as *Pleioblastus* bamboo and *Miscanthus* silver grass (Suppl. material 1: table S1). The nests of the new species were not collected from urbanized areas such as open parks, which are typical habitats for tramp species. On Amami-ôshima Island, the new species was collected at a significantly higher altitude (50–365 m, *N* = 27) than *M. intrudens* which was found at lower elevations (3–165 m, *N* = 8), as indicated in the Mann–Whitney U-test (Z = −3.6684, *p* = 0.0002). The number of workers per nest ranged from 273 to 3861 in the new species, with a mean ± SD of 1571.4 ± 985.2 (*N* = 25, Suppl. material 1: table S1). In the case of *M. intrudens*, the number of workers in a nest varied from 65 to 3196, with a mean ± SD of 578.9 ± 614.7 (*N* = 39, Suppl. material 1: table S1). The Mann–Whitney U-test revealed that the mean number of workers per nest in the new species was significantly higher than in *M. intrudens* (Z = −4.5342, *p* < 0.0001, Fig. 4). Meanwhile, there was no significant difference in the number of the queens per nest between the new species (9.1 ± 7.3, range 1–27, *N* = 25, Suppl. material 1: table S1) and *M. intrudens* (7.2 ± 6.3, range 1–26, *N* = 39, Suppl. material 1: table S1) (Mann–Whitney U-test, Z = −1.0908, *p* = 0.2753).

### ﻿Morphological comparisons

**Figure 5.**
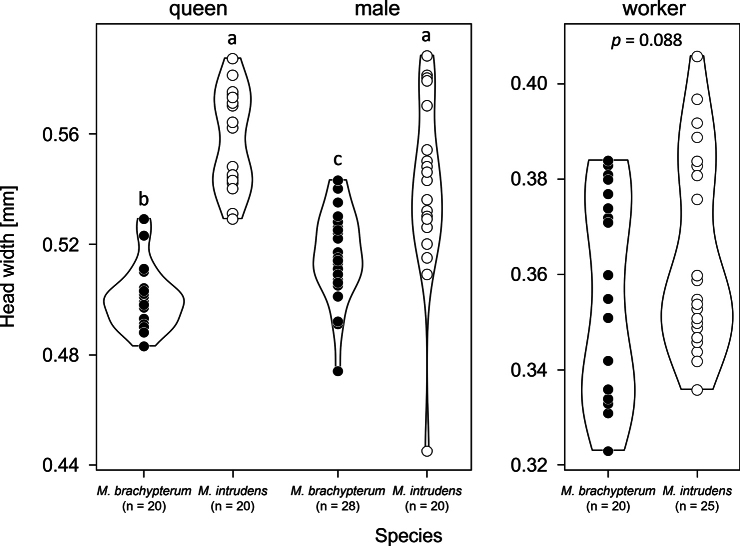
Head width of each caste of *M. brachypterum* and *M. intrudens*. Different letters (a-c) indicate significant difference at *p* = 0.05 level using the Mann–Whitney U-test.

Morphological measurements revealed that the queens of *M. brachypterum* sp. nov. showed a distinctly small head width (mean ± SD: 0.50 ± 0.01 mm, range 0.48–0.53 mm, *N* = 20) compared to *M. intrudens* (mean ± SD: 0.56 ± 0.02 mm, range 0.53–0.59 mm, *N* = 20), with no overlap (Mann–Whitney U-test with Holm correction, Z = 5.399, *p* < 0.0001, Fig. 5). Also in the males, the head width was significantly smaller in the new species (mean ± SD: 0.52 ± 0.02 mm, range 0.47–0.54 mm, *N* = 28) than in *M. intrudens* (mean ± SD: 0.54 ± 0.03 mm, range 0.44–0.59 mm, *N* = 20), but the difference was marginal (Z = 3.556, *p* = 0.0007). In both species, the head width of queens and males overlaps. However, *M. brachypterum* males were slightly larger than queens (Z = 3.756, p = 0.0007), whereas no significant sexual dimorphism was found in *M. intrudens* (Z = −1.732, p = 0.0833). In workers, head width of the newly described species (mean ± SD: 0.35 ± 0.02 mm, range 0.32–0.38 mm, *N* = 20) did not differ significantly from that of *M. intrudens* (mean ± SD: 0.36 ± 0.02 mm, range 0.34–0.41 mm, *N* = 20), with considerable overlap between the two species (Z = 1.703, *p* = 0.0885; Fig. 5).

**Figure 6.**
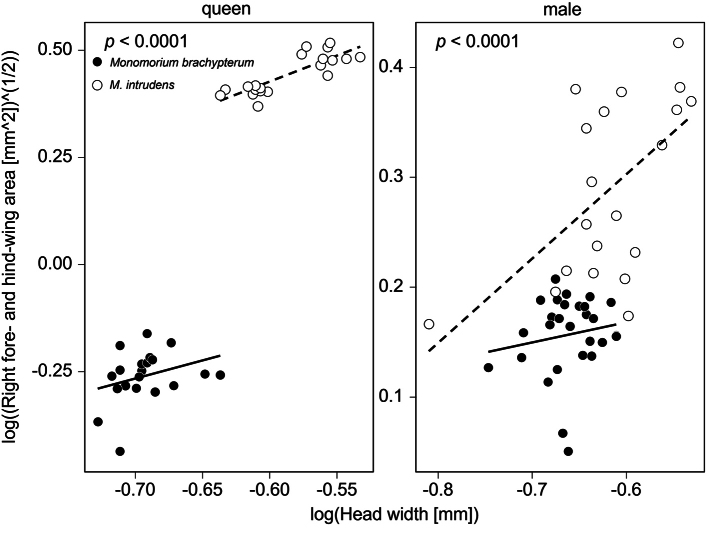
Relationship between the head width and wing area of *Monomorium brachypterum* and *M. intrudens*. The solid and dashed lines are the regression lines for *M. brachypterum* and *M. intrudens*, respectively.

Degeneration in wings was observed in the queens of *M. brachypterum* sp. nov. (*M. intrudens* has fully developed wings). In both the right forewing (Mann–Whitney U-test, queen: Z = 5.411, *p* < 0.0001, male: Z = 5.292, *p* < 0.0001) and right hindwing (queen: Z = 5.410 *p* < 0.0001, male: Z = 4.894, *p* < 0.0001), the wing area of the new species was significantly smaller than that of *M. intrudens*. The results of the ANCOVA showed that the newly described species exhibited significantly smaller wing sizes compared to *M. intrudens* in both the queens (Estimate = −0.58775, SE = 0.03271, t = −17.967, *p* < 0.0001; Fig. 6) and the males (Estimate = −0.08748, SE = 0.02009, t = −4.355, *p* < 0.0001). Notably in queens, the average total wing area (= forewing and hindwing) of *M. brachypterum* (mean ± SD: 0.6 ± 0.07 mm^2, range 0.42–0.72 mm^2, *N* = 20) was considerably smaller than that of *M. intrudens* (mean ± SD: 2.44 ± 0.22 mm^2, range 2.09–2.81 mm^2, *N* = 20), suggesting that the wings are no longer functional. In both queens and males, the logarithm of head width had a significant positive effect on wing size. In queens, the effect was stronger (Estimate = 1.08701, SE = 0.27264, t = 3.987, p = 0.0003), while in males, the effect was weaker but still statistically significant (Estimate = 0.43033, SE = 0.193, t = 2.23, p = 0.0303). The model showed a good fit for both the queens (R^2^ = 0.9844, F (2, 37) = 1168, *p* < 0.0001) and the males (R^2^ = 0.4688, F (2, 50) = 22.06, *p* < 0.0001).

**Figure 7.**
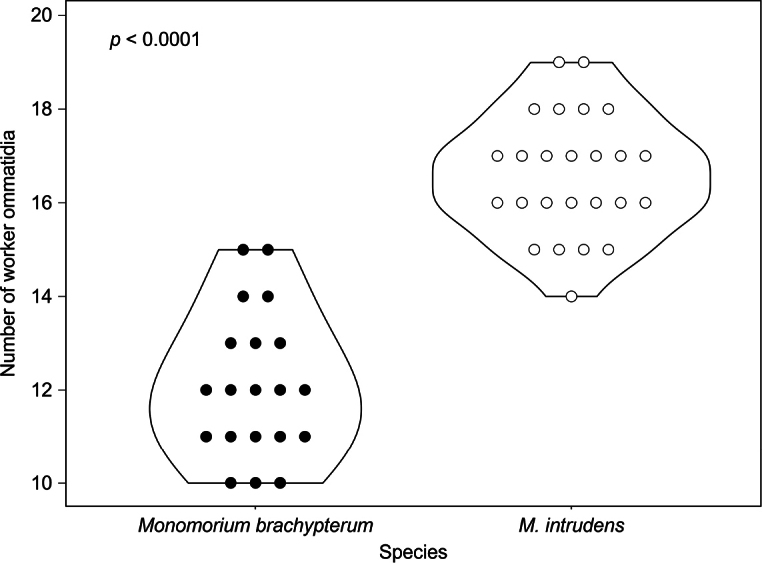
The number of ommatidia of workers of *Monomorium brachypterum* and *M. intrudens*.

In workers, *M. brachypterum* had significantly fewer ommatidia (mean ± SD: 12.1 ± 1.55, range 10–15, *N* = 20) than *M. intrudens* (mean ± SD: 16.6 ± 1.29, range 14–19, *N* = 25), as compared using the Mann–Whitney U-test (Z = 5.5893, *p* < 0.0001; Fig. 7). The thorax widths of the new species were also smaller than that of *M. intrudens* (Mann–Whitney U-test, Z = 2.8226, *p* = 0.0048) in the workers. In the ANCOVA, the results showed a good fit (R^2^ = 0.9241, F (2, 42) = 255.8, *p* < 0.0001) in both species, with the logarithm of head width having a significant effect on the logarithm of thorax width. Specifically, the new species exhibited a significantly smaller thorax width compared to *M. intrudens* (Estimate = −0.034965, SE = 0.005996, t = −5.831, *p* < 0.0001). Additionally, the log of the head width had a highly significant positive effect on the thorax width (Estimate = 1.040618, SE = 0.052408, t = 19.856, *p* < 0.0001).

In the comparison of populations of *M. brachypterum* and *M. intrudens* from sympatric regions, interspecific differences in male head width and worker thorax width were no longer statistically significant. In contrast, clear interspecific differences in other external morphological traits remained consistent even when the analysis was restricted to sympatric populations (Table 2). Comparison data including all populations are summarized in Suppl. material 9: table S3.

**Table 2.** Morphological comparison between *Monomorium brachypterum* and *M. intrudens* in overlapping populations, using the Mann–Whitney U-test statistic.

male	M. brachypterum (N = 29)				M. intrudens (N = 10)				Z score	p-value
	mean	sd	min	max	mean	sd	min	max		
head width [mm]	0.52	0.02	0.47	0.54	0.53	0.03	0.44	0.55	1.957	0.05036
thorax width [mm]	0.46	0.03	0.41	0.52	0.44	0.03	0.40	0.48	-1.190	0.2339
forewing area [mm^2]	1.07	0.08	0.85	1.18	1.20	0.08	1.09	1.32	3.796	0.0001468
hindwing area [mm^2]	0.30	0.02	0.23	0.34	0.34	0.03	0.31	0.38	3.185	0.001448
queen	M. brachypterum (N = 20)				M. intrudens (N = 10)				Z score	p-value
	mean	sd	min	max	mean	sd	min	max		
head width [mm]	0.50	0.01	0.48	0.53	0.55	0.01	0.54	0.57	4.403	1.07E-05
thorax width [mm]	0.38	0.01	0.36	0.41	0.43	0.02	0.41	0.47	4.381	1.18E-05
forewing area [mm^2]	0.39	0.04	0.27	0.46	1.87	0.20	1.62	2.17	4.402	1.07E-05
hindwing area [mm^2]	0.21	0.03	0.15	0.27	0.54	0.06	0.47	0.64	4.400	1.08E-05
worker	M. brachypterum (N = 20)				M. intrudens (N = 15)				Z score	p-value
	mean	sd	min	max	mean	sd	min	max		
head width [mm]	0.35	0.02	0.32	0.38	0.36	0.01	0.34	0.38	0.501	0.6166
thorax width [mm]	0.21	0.01	0.20	0.24	0.22	0.01	0.21	0.24	1.585	0.113
number of ommatidia	12.10	1.55	10	15	16.67	1.23	15	19	4.932	8.13E-07
thorax/head ratio	0.60	0.01	0.56	0.63	0.62	0.01	0.60	0.63	3.800	0.0001445

## ﻿Taxonomy

### ﻿Key to the named Japanese species of *Monomorium* (worker) (partly after Terayama et al. 2022)

We have examined many worker samples from colonies for most species studied, which are basically monomorphic. Size and color pattern variations are small in each species, and species-level identification is, in most cases, possible based on the size and coloration, while among smaller species, useful structural characters (including pilosity) are few and trivial, and often show variation in each species.

**Table:** 

1	Head dorsum often with sparse minute punctures, always smooth and shiny. Lateral face of mesosoma extensively smooth and shiny; sculpture, if any, restricted to small areas. Body generally small, with total length c. 1.5 mm and head width 0.3–0.4 mm	**2**
–	Head dorsum densely sculptured and matte. Lateral face of mesosoma extensively sculptured and matte. Body larger with total length more than 1.7 mm, generally more than 2.0 mm, and head width 0.4–0.7 mm	**7**
2	Body dark brown, or bicolorous with mesosoma and waist yellow to orange and head or gaster, or both, extensively dark (Fig. 11A–D). Flagellomere 8 (antennomere 10) as long as or longer than broad (Fig. 12A). Propodeal dorsum, petiole and postpetiole each generally with 2 or more pairs of standing hairs (Fig. 12C)	**3**
–	Body predominantly yellowish (in *M. hiten* gastral tergite 1 laterally with pair of large dark markings) (Fig. 11E, F). Flagellomere 8 broader than long (Fig. 12B). Propodeal dorsum, petiole and postpetiole each generally with only one pair of standing hairs; petiole or postpetiole occasionally with additional pair of short hairs (Fig. 11D, E)	**6**
3	Body unicolorous, nearly entirely dark brown (discolored if kept in alcohol for a long time) (Fig. 11A). Frontal lobe longitudinally striate (Fig. 12F). Anterolateral corner of median disc of clypeus distinctly angulate (Fig. 12H)	***M. chinensis* (? tramp)**
–	Body bicolorous; mesosoma and waist always yellowish/orangish (Fig. 11B–D). Frontal lobe not striate with outer margin carinate (Fig. 12G). Anterolateral corner of median disc of clypeus indistinct or rounded (Fig. 12I, J)	**4**
4	Head and gaster dark brown to blackish (Fig. 11D). Hairs on antennal scape appressed or strongly decumbent (Fig. 12K). Anterior or lower portion, or both, of mesopleuron often with sculptured area(s); lower portion of metapleuron usually striate	***M. floricola* (tramp)**
–	Head yellowish brown; gaster dark brown, often with paler areas of various sizes (Fig. 11B, C). Hairs on antennal scape usually suberect, never appressed (Fig. 12L). Mesopleuron and metapleuron predominantly smooth	**5**
5	Pale marking on gastral tergite 1 restricted to basal small area; other tergites dark brown (Fig. 11B). Eye generally with 7 or more ommatidia along its longest axis	***M. intrudens* (native)**
–	Pale marking(s) on gastral tergite 1 more extensive; other tergites often with pale areas (Fig. 11C). Eye with 5–6 ommatidia along its longest axis	***M. brachypterum* sp. nov. (native)**
6	Gastral tergite 1 yellow, laterally with paired dark spots that are rather clearly defined (Fig. 11F). Area between eye and anterior margin of cranium (malar space) extensively rugulose. Postpetiole with pair of long erect hairs on its dorsum, only rarely with additional pair (Fig. 12D)	***M. hiten* (native)**
–	Gastral tergite 1 yellow, without such well-defined spots, but often laterally and/or posteriorly tinged with brown (Fig. 11E). Area between eye and anterior margin of cranium extensively smooth; rugulae confined to anterior zone. Postpetiole often with two pairs of erect hairs of different lengths (Fig. 12E)	***M. triviale* (native)**
7	Promesonotum with two pairs of erect hairs. Gastral tergites brown, but first tergite with variable size of pale areas	***M. pharaonis* (tramp)**
–	Promesonotum without erect hairs. Gastral dorsum blackish	**8**
8	Postpetiole distinctly broad, 1.2 times as broad as petiole in dorsal view, as high as petiole in profile view. Total body length c. 1.7 mm	***M. sahlbergi* (tramp)**
–	Postpetiole nearly as broad as petiole in dorsal view, lower than petiole in profile view. Body much larger, measuring 3.0–3.5 mm in total length	***M. salomonis* (tramp)**

## ﻿Description

### ﻿ Monomorium brachypterum

Taxon classificationAnimaliaHymenopteraFormicidae

Idogawa & Yamane sp. nov.

8F517AFC-F8B2-5CA2-B5B3-5A3366D662DB

https://zoobank.org/6427F3CB-BEDC-4A05-B071-E446800DAA39

#### Type material.

***Holotype***: • queen, Yuwan, Amami-ôshima, the Central Ryukyu Islands, Japan, 29.III.2023, N. Idogawa leg. (unique specimen identifier: KMNHSKYC25-01), deposited in SKYC (Kitakyushu Museum of Natural History and Human History: KMNH). ***Paratypes***: • 6 dealated queens (KMNHSKYC25-02–07), 30 workers (KMNHSKYC25-08–37) and 7 males (KMNHSKYC25-37–43), the same data as the holotype, deposited in SKYC and KUEC (Institute of Tropical Agriculture, Kyushu University).

#### Non-type material examined.

***Kyushu mainland***: • 13 workers & 4 dealate queens, Eboshi-dake, Kagoshima-shi, 24.ii.2018, dead sasa bamboo, E. Yamane leg., JP18-SKY-27. ***Amami-ôshima***: • 14 workers & 10 dealate queens, Arimori-jinja, Uragami, Naze, 8.x.2022, Sk. Yamane leg., JP22-SKY-126; • 16 workers & 9 dealate queens, same loc. & collector, 8.x.2022, dead bamboo, JP22-SKY-127; 5 workers, Sutarumata, Sumiyô, 31.vii.2021, dead stem, Sk. Yamane leg., JP21-SKY-149; • 14 workers & 3 dealate queens, same data as above, JP21-SKY-150; • 1 worker, Yakukachi-gawa Eco-road, Sumiyô, 29.vii.2021, *Mallotus* EFN, Sk. Yamane leg.; • 3 workers & 2 dealate queens, Yuwan-dake, 7.vi.2018, S. Fukumoto leg.; • 2 workers, Mt. Yui-dake, Setouchi, 20.iii.2019, Y. Hisasue leg. ***Okinawa-jima***: • 10 workers, Ishikawa-Sonan, Uruma, 29.i.2023, S. Araki leg., Msp20230129_01; • 10 workers & 2 dealate queens, Imadomari, Nakijin, Kunigami, 10.vii.2023, N. Idogawa leg., Msp20230710_01; • 10 workers & 2 dealate queens, Oku, Kunigami, 04.ix.2023, N. Idogawa leg., Msp20230904_01.

#### Description.

**Worker** (Fig. 10). ***Measurements*** (*N* = 6). TL 1.68–2.00, HW 0.32–0.38, HL 0.39–0.45, EW 0.04–0.06, EL 0.06–0.07, SL 0.27–0.31, PrW 0.21–0.23, PtW 0.10–0.11, PptW 0.11–0.12. Indices. ***Indices*.**CI 80–85, SI 77–85.

***Structure*.** Head in full-face view, little longer than broad, with very feebly concave posterior margin and shallowly convex lateral margin. Frontal lobe small; its outer margin weakly convex and only partly covering antennal socket; frontal carinae very short, strongly pigmented and diverging posteriad. Clypeus posteriorly demarcated from frons with fine line; its median portion with straight anterior margin. Eye located anterior to midlength of head and slightly breaking lateral margin of head, oblong in shape, relatively small with maximum diameter 0.16× – 0.20× HW and 5–6 ommatidia in longest row. Antennal scape, when laid straight back from its insertion, not reaching posterior margin of head. With mesosoma in profile view, dorsal outline of promesonotum convex, sloping posteriad to metanotal groove; propodeal dorsum short, continuing to posterior slope (declivity). With mesosoma in profile view mesopleuron separated from lateral face of pronotum with sharp furrow and from metapleuron with cross-rimmed furrow, which is continuation of dorsal metanotal groove. Propodeum completely fused with metapleuron; distance between metapleural gland bulla and propodeal spiracle generally as long as or shorter than spiracle diameter. With waist in dorsal view, petiole node distinctly broader than long; postpetiole node as broad as and longer than petiole node. With waist in profile view, petiole node subtriangular, with rounded apex; sternite of petiole ventrally weakly convex; postpetiole node globular, much lower than petiole node.

***Sculpture***. Body extensively smooth and shiny with scattered small piligerous punctures over dorsum of body. Lateral portion of cranium along anterior margin (just above lateral lobe of clypeus) longitudinally striate. Clypeus largely smooth, but lateral slope of its main disc punctate; transverse lateral portion (lobe) above mandibular base irregularly sculptured. Antennal scape essentially smooth, but pedicel and flagellum densely covered with minute sculpture. Mesosoma essentially smooth, but metanotal groove with several longitudinal carinae. Anterior slopes of petiole and postpetiole with relatively large punctures, but highly shiny. Gastral segments essentially smooth.

***Pilosity*.** Cephalic surface with relatively sparse erect hairs; posterolateral corner of head with several pairs of erect hairs; antennae with erect hairs, which are as long as diameter of scape or a little shorter. Promesonotum dorsally with at least three pairs of erect hairs, propodeum with single pair; petiole and postpetiole each with two to three pairs of backward-directed hairs. First gastral tergite and sternite with several pairs of erect hairs; pilosity of remaining gastral tergites restricted to posterior margins.

***Coloration*.** Bicolored. Head, mesosoma, petiole, postpetiole and appendages yellowish. Gaster pale to dark brown; first gastral tergite with basal yellowish marking, which is variable in size and often extensive; second tergite sometimes partly yellowish; lateral portion of these tergites almost always darker than median portion.

**Figure 8.**
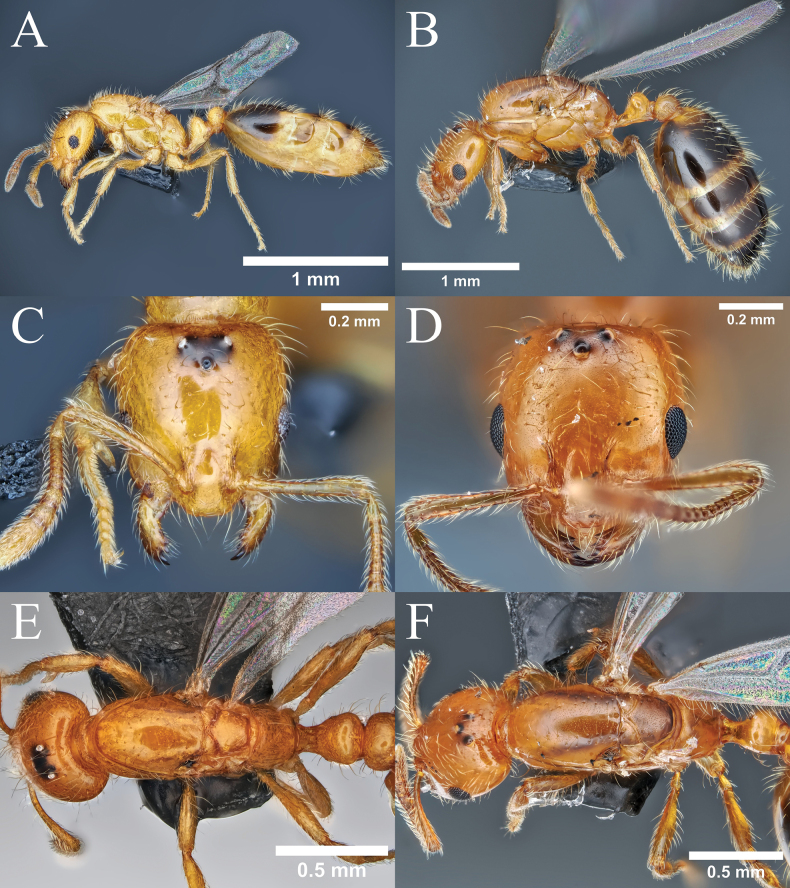
Queen morphology of *Monomorium* species. **A.***M. brachypterum* sp. nov., body in profile view; **B.***M. intrudens*, body in profile view; **C.***M. brachypterum* sp. nov., head in full-face view; **D.***M. intrudens*, head in full-face view; **E.***M. brachypterum* sp. nov., thorax in dorsal view; **F.***M. intrudens*, thorax in dorsal view.

**Queen** (Fig. 8). ***Measurements*** (*N* = 6). TL 3.07–3.63, HW 0.46–0.51, HL 0.52–0.58, EW 0.08–0.09, EL 0.11–0.13, SL 0.35–0.41, PrW 0.35–0.38, PtW 0.19–0.24, PptW 0.23–0.27. ***Indices***. CI 87–93, SI 71–81.

***Structure*.** Head in full-face view longer than broad, with very feebly concave posterior margin and shallowly convex lateral margin, slightly narrowed anteriorly. Frontal lobe small, anteriorly roundly convex outward, only partly covering antennal socket; frontal carina short extending up to posterior margin of ill-defined antennal scrobe. Clypeus roughly divided into posterior narrow area between frontal lobes, median disc and transverse lateral lobe; posterior area distinctly narrowed posteriad; median disc with convex anterior margin, of which median portion almost straight. Mandible nearly parallel-sided, apically inwardly curved, with four teeth on masticatory margin; apical tooth largest and basal smallest; basal margin entire. With head in full-face view, ocelli arranged in low triangle; lateral (posterior) ocelli separated from posterior margin of head by diameter of ocellus. Eye located distinctly anterior to midlength of head, barely breaking lateral margin of head in full-face view, with maximum diameter 0.22× – 0.25× HW; distance between anterior eye margin and anterior margin of cranium (malar space) slightly shorter than maximum eye diameter. Antennal scape, when laid straight back, not reaching posterior head margin; pedicel long, more than twice as long as its width, longer than first to third flagellomeres combined. Mesosoma in dorsal view elongate, much narrower than head, slightly narrowed posteriad. Mesonotum occupying most of pro-mesonotal dome, leaving anterior and lateral small portions of pronotum visible from above; mesoscutum largest, longer than broad; scuto-scutellar sulcus posteriorly deeply incised in middle; scutellum much narrower than scutum, slightly longer than broad. Metanotum transverse, clearly separated anteriorly and posteriorly with sharp furrows. Propodeum with dorsum short and not clearly demarcated from declivity. With mesosoma in profile view, its dorsal outline very shallowly convex but anterior slope of pronotum rather steep. Pronotum large, slightly widened posteriad, clearly demarcated from mesopleuron. Mesopleuron large, as long as high, divided into upper and lower sections with sharp furrow. Metapleuron demarcated from mesopleuron with sharp furrow, but from propodeum only in upper portion with broad furrow, which is cross-rimmed; lower portion fused with propodeum. Propodeum with dorsal outline almost evenly curved and sloping, with no distinction between dorsum and declivity; propodeal spiracle round, separated from upper margin of metapleural gland bulla by less than spiracle diameter; metapleural lobe small and low. With waist in dorsal view, petiole broader than long, roundly convex laterally; postpetiole broader than petiole, with narrow ring-like cinctus; in both petiole and postpetiole dorsum not clearly defined, only recognized by its sculpture (smooth). With waist in profile view, petiole with short peduncle, which is slightly higher than long; node apically broadly round with anterior slope longer and gentler than posterior slope; ventral margin of sternite almost straight to very shallowly convex, often lamellate; subpetiolar process absent; postpetiole lower than petiole, rather globular, with broadly rounded apex. Gaster in dorsal view elongate, with parallel to shallowly convex outer margins; anterior margin of tergite 1 broadly concave with anterolateral corner distinctly angulate to acute apically; cinctus widened dorsally, cross rimmed, not clearly demarcated from posttergite.

***Sculpture*.** Body extensively smooth and shiny. Dorsum of head sparsely with small piligerous punctures, shiny; venter of head essentially smooth. Frontal lobe longitudinally striate; area between eye and mandibular base/clypeus longitudinally striate. Clypeus with smooth posterior area, superficially sculptured median disc, and densely sculptured and dull lateral zone. Mandible finely striate in basal half, nearly smooth in apical half. Mesosoma almost entirely smooth and shiny; lower portions of metapleuron and lateral face of propodeum striate; metapleural gland bulla entirely striate. Petiole extensively smooth, with peduncle and sternite finely sculptured; postpetiole with dorsum smooth; other parts sculptured. Gaster almost entirely smooth and highly shiny; cinctus of first gastral segment dorsally longitudinally rugose; pretergite of the segment minutely transversely striate.

***Pilosity*** Cephalic dorsum including clypeus with many erect hairs of varying length; longest hairs longer than major diameter of eye; venter of head with shorter hairs. Mandible with suberect to decumbent hairs. Antennomeres with many suberect to decumbent hairs of relatively uniform length; some hairs on scape as long as diameter of scape or a little shorter; hairs on apical flagellomere much shorter and very dense. Promesonotum dorsally with many erect hairs. Anterior slope of petiole bearing relatively short slanting hairs; dorsum of petiole with a few rather long curved hairs; anterior slope and dorsum of postpetiole with similar hairs; venter of waist without hairs except for extreme anterior portion of petiolar sternite that is covered with minute hairs. Gastral tergites with many long erect hairs; hairs on sternites slightly shorter. Legs densely with short decumbent to appressed hairs.

***Coloration*.** Head, antenna, mesosoma, waist and legs yellow or yellowish orange. Gastral coloration highly variable; in a few specimens all tergites extensively brown, but in most cases first tergite with anterior and posterior yellowish bands and other tergites with yellowish areas; generally, sternites much paler, often entirely yellowish. Vertexal area surrounded by ocelli dark brown to black.

***Wings*.** Both fore- and hindwings reduced in size and venation. Forewing not reaching midpoint of gaster; pterostigma reduced; basal cell closed; subbasal cell incompletely closed; first and second submarginal cells incompletely closed; other cells absent. Hindwing with much reduced venation, without distinctly closed cell.

**Figure 9.**
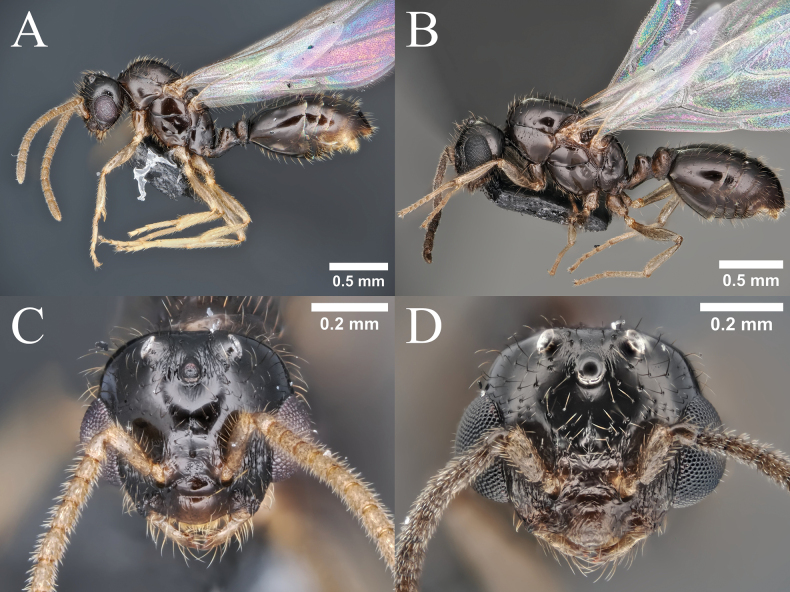
Male morphology of *Monomorium* species. **A.***M. brachypterum* sp. nov., paratype male, body in profile view; **B.***M. intrudens*, body in profile view; **C.***M. brachypterum* sp. nov., head in full-face view; **D.***M. intrudens*, head in full-face view.

**Male** (Fig. 9). ***Measurements*** (*N* = 6). TL 2.62–2.89, HW 0.47–0.51, HL 0.46–0.51, EW 0.17–0.19, EL 0.20–0.24, SL 0.15–0.16, PrW 0.42–0.44, PtW 0.18–0.21, PptW 0.22–0.24. ***Indices***. CI 98–10.6, SI 29–34.

***Structure*.** Head in full-face view as long as broad excluding eyes, round, with convex posterior margin (but narrowly concave between lateral ocelli) and round posterolateral corner; vertex and temple continuous with smooth curve; occipital carina present only behind vertex, not extending to lateral face of cranium. Frontal lobe narrow so that antennal socket is largely exposed; frontal carinae parallel, weak, short, extending posteriad only for length of diameter of antennal socket. Clypeus roughly divided into three parts, i.e., transverse basal portion, of which posterior margin vaguely defined and barely reaching level of midlength of antennal socket, large median disc with lateral slope, and transverse anterolateral portion; median disc demarcated from basal portion by fine but distinct line; median disc with shallowly convex anterior margin in dorsal view, and convex dorsal margin in profile. Eye large, strongly bulging, with maximum diameter 0.41× – 0.49× HW; lateral ocellus located close to posterior margin of head in full-face view; median ocellus smaller than lateral ocellus, with its diameter less than half the diameter of lateral ocellus, sometimes much reduced. Mandible narrow with three teeth. Antenna with 11 flagellomeres (13 antennomeres); scape, when laid straight back from its insertion, not reaching level of posterior eye margin; pedicel slightly longer than and broader than first flagellomere; flagellomeres each longer than broad; apical flagellomere twice as long as broad. Mesosoma in dorsal view much narrower than head, elongate, broadest at wing bases; in profile 1.9× as long as high. Pronotum mostly invisible; anterior lobe (neck) short (transverse). Mesonotum occupying most of thoracic dorsum; scuto-scutellar sulcus posteriorly deeply concave, anteriorly demarcated with sharp and deep furrow; mesoscuttelum slightly longer than broad. Metanotum distinctly transverse, flanked by sharp furrow anteriorly and posteriorly. Propodeum almost without dorsal face. With mesosoma in profile view, pronotum demarcated from mesonotum and mesopleuron, ventrally carinate. Mesopleuron divided into upper and lower sections with sharp furrow, demarcated from metapleuron with distinct furrow; upper section with short furrow under forewing base. Metapleuron separated from propodeum in upper portion, but completely fused with propodeum in lower portion. Propodeum with dorsal outline almost straight; dorsal and posterior (declivity) slope completely continuous. Petiole in dorsal view with its node much broader than long, in profile view with distinct anterior peduncle, longer than high with apically weakly rounded node; anterior slope of node much longer than posterior slope; ventral outline straight. Postpetiole in dorsal view broader than petiole, much broader than long, in profile view as high as petiole, with anterior and posterior margins rather parallel; subpostpetiolar process distinct, with sharp corner. Gaster elongate; first gastral segment distinctly narrowed anteriad; cinctus present, consisting of chain of irregular punctures; pretergite convex posteriorly.

**Figure 10.**
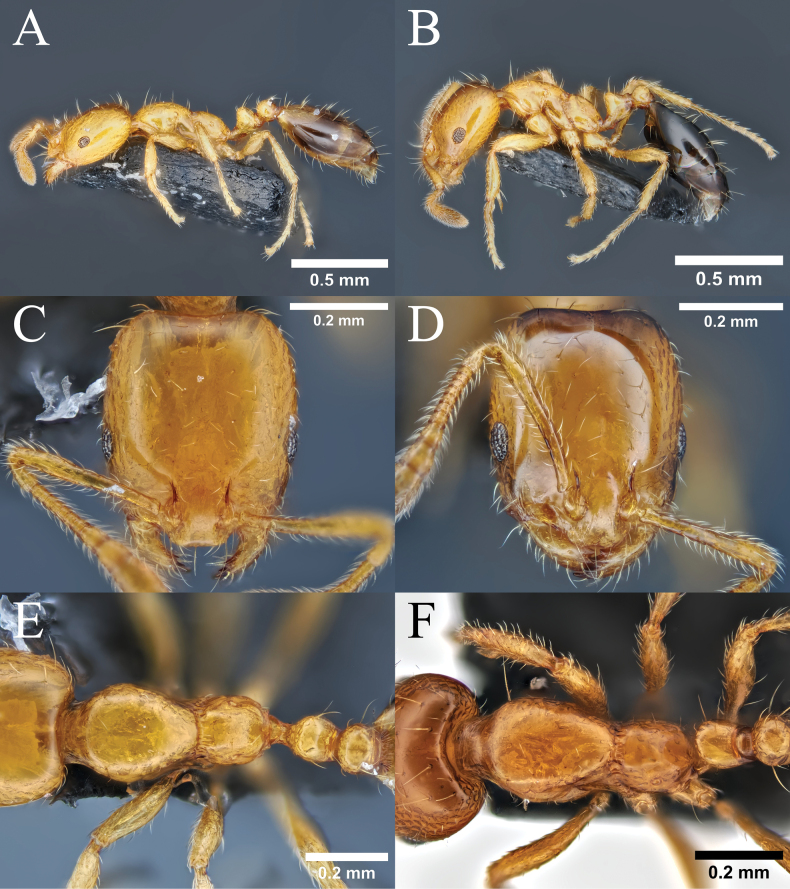
Worker morphology of *Monomorium* species. **A.***M. brachypterum* sp. nov., paratype worker, body in profile view; **B.***M. intrudens*, body in profile view; **C.***M. brachypterum* sp. nov., head in full-face view; **D.***M. intrudens*, head in full-face view; **E.***M. brachypterum* sp. nov., thorax in dorsal view; **F.***M. intrudens*, thorax in dorsal view.

***Sculpture*.** Body extensively smooth and shiny. Area between antennal socket and eye with parallel longitudinal rugulae. Ocellar area longitudinally striate. Antennal scape and pedicel essentially smooth; flagellum almost entirely superficially sculptured and weakly shiny. Peduncle, posterolateral portion and anterior face of petiole sculptured; postpetiole laterally and posteriorly sculptured.

**Figure 11.**
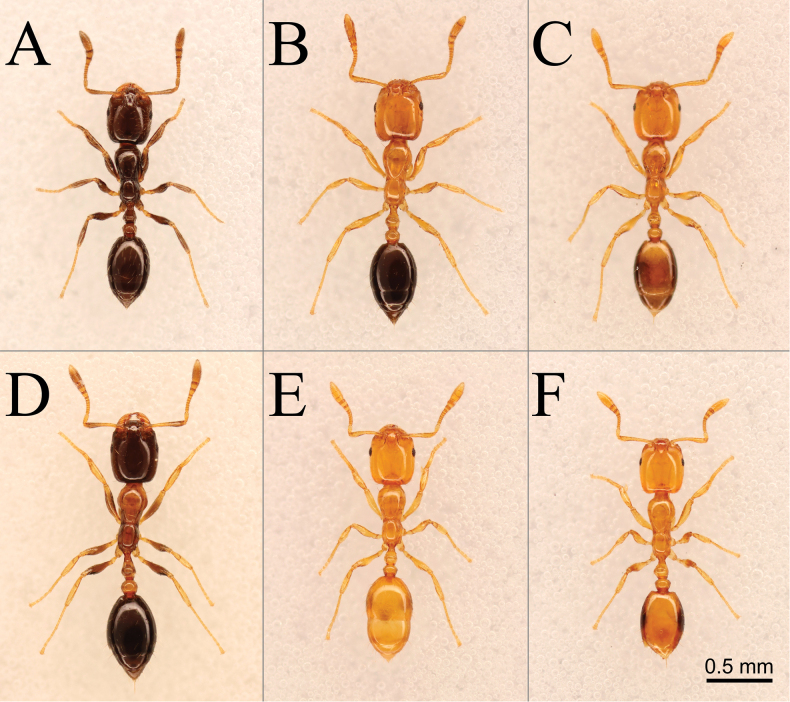
Dorsal views of small shiny *Monomorium* species from Japan. **A.***M. chinense*; **B.***M. intrudens*; **C.***M. brachypterum*; **D.***M. floricola*; **E.***M. triviale*; **F.***M. hiten*.

***Pilosity*.** Dorsum of body with relatively sparse, erect and slanting hairs over the surface. Antennal scape on dorsal face with relatively long suberect hairs, some of which are as long as scape diameter; hairs on anterior and ventral faces shorter and denser; pedicel and flagellum with dense suberect to decumbent short hairs. Eye with erect hairs of moderate density. Petiole with dense suberect/decumbent hairs on anterior slope, but without erect hairs on posterior face; postpetiole with a few erect hairs on posterior face and much shorter hairs ventrally. Femora of legs with relatively long suberect/decumbent hairs on dorsal and ventral faces; tibiae densely with much shorter decumbent/appressed hairs; all hairs on tarsi still shorter and near-appressed. Hairs on venter of gaster similar to those on its dorsum.

**Figure 12.**
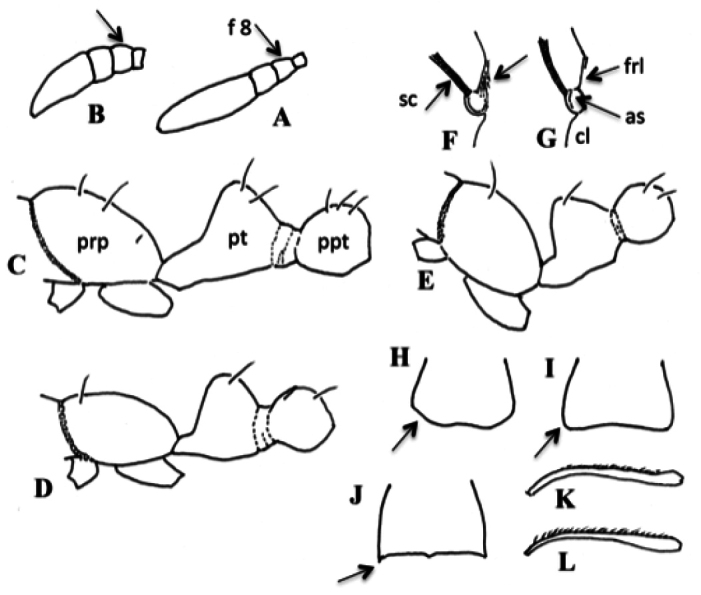
Key features of small shiny *Monomorium* species from Japan. **A, B.** Apical flageromeres of worker antenna, showing relative length of flagellomere 8 (antennomere 10): **A.***Monomorium hiten*; **B.***M. floricola*, f8: flagellomere 8. **C–E**. Pilosity on propodeum and waist (worker): **C.***Monomorium floricola*; **D.***M. hiten*; **E.***M. triviale*. Abbreviations: prp: propodeum, pt: petiole, ppt: postpetiole; **F, G.** Sculpture on frontal lobe (worker): **F.***Monomorium chinense*; **G.***M. floricola*. Abbreviations: as: antennal socket, sc: antennal scape, cl: clypeus, frl: frontal lobe; **H–J.** Median disc of clypeus (worker), showing condition of ita anterolateral corner (arrow): **H.***Monomorium brachypterum* sp. nov.; **I.***M. floricola*; **J.***M. chinense*; **K, L.** Pilosity on antennal scape (worker): **K.***Monomorium floricola*; **L.***M. intrudens*.

***Coloration*.** Body dark reddish brown to blackish; mesosoma slightly lighter than head and gaster. Antenna and mandible light brown. Legs brown to yellowish, with tibiae pale yellow.

***Wings*.** Forewing with distinct pterostigma; costal and subcostal veins close to each other; basal, marginal and first submarginal cells closed; second submarginal cell incompletely closed; first discal cell absent; subbasal and second discal cells not separated. Hind wing with much reduced venation, without distinctly closed cell.

#### Remarks.

Among Japanese congeneric species, *Monomorium brachypterum* sp. nov. is very similar in morphology and body coloration to the most closely related *M. intrudens*. Compared with *M. intrudens*, this species is relatively smaller in all sexes and castes. In the queens, the new species can be easily recognized by body size (no overlapping in head width) and coloration of the gastral tergites from *M. intrudens* (in *brachypterum* tergites generally brownish with more extensive yellowish markings). Furthermore, the dorsal/declivity outline of the propodeum in profile view is less convex and completely evenly curved throughout in the new species, while it is more convex and with a steeper declivity in *M. intrudens*.

In the worker, distinction of the two species is more difficult because the body size as expressed by head width overlaps between them. However, *Monomorium brachypterum* sp. nov. can be separated from *M. intrudens* by: 1) smaller eye (number of ommatidia 10–15 in *M. brachypterum* vs. 15–18 in *M. intrudens*, but exceptions present in both species), and 2) gastral tergites pale brown; first tergite basally and apically with yellowish areas of variable size and other tergites often extensively yellowish (gastral tergites dark brown; yellowish marking confined to small basal area of first tergite in the latter). In both female castes, the body color of the new species tends to be paler than that of *M. intrudens*, but further research is needed to explore intraspecific variation in body coloration for both species.

The male of *M. brachypterum* is easily distinguished from that of *M. intrudens* by the following set of characteristics: 1) body constantly smaller with head width 0.47–0.51 mm (0.51–0.55 mm in the latter), 2) median ocellus much reduced and sometimes vestigial, its diameter always less than half the diameter of lateral ocellus (median ocellus normal in the latter), 3) antennal pedicel longer and broader than first flagellomere (shorter than and as broad as first flagellomere in the latter), and 4) antenna light brown to brown (generally entirely dark brown; at least apical 3 flagellomeres very dark).

#### Etymology.

The species name *brachypterum* (from Greek “*brachys*,” meaning “short,” and “*pteron*,” meaning “wing”) is treated as a Latinized neuter adjective in the nominative singular, referring to the reduced wings of the queen.

## ﻿Discussion

In this study, a new *Monomorium* species, *M. brachypterum* sp. nov. is described and the detailed phylogenetic tree of the Japanese *Monomorium* species is provided with the newly assembled complete mitochondrial genomes of five species. Together with the three artificially transported species previously reported by Terayama et al. (2022), this study adds a tenth species to the checklist of Japanese *Monomorium*. Although Teranishi (1940) described three varieties of *M. intrudens* (M. intrudens var. gracilum , var. robustum and var. satoi), we follow Ogata and Bolton (1989) in treating them as junior synonyms of *M. intrudens*, as we agree with their statement that “the distinguishing characters mentioned by him are very minor.”

The crucial issue is whether our concept of *M. intrudens* is correct or not; if incorrect, all our reasoning for establishing a new species would become invalid. In the original description of *M. intrudens* (worker), the gastral color pattern is described as ‘the extreme base of the abdomen pale, the rest of it nearly black’ (this pattern is also confirmed in the image of the type material CASENT0902283, Antweb, http://www.antweb.org, accessed 29 May 2025). Furthermore, the type locality of *M. intrudens* is Hyogo Prefecture, Honshu, Japan, where only samples with the typical color pattern of *M. intrudens* have been found to date. All this shows that our concept of *M. intrudens* is correct. The species is widely distributed with the gastral color pattern being rather stable throughout its range, while the range of the new species is confined to southern Japan (South Kyushu and the Ryukyu Islands).

The situation has been further complicated by the proposal of several infraspecific forms for *M. intrudens*. Three varieties were described by Teranishi (1940) after his death, based mainly on the queen caste with brief and incomplete descriptions. All these forms were synonymized by Ogata and Bolton (1989) with *M. intrudens*. Together with the unfortunate loss of the type specimens during World War II, it is almost impossible to determine the relationship between these invalid forms and *M. brachypterum* sp. nov. However, our careful examination of the original descriptions of these forms has shown that the new species is different from these forms in color pattern, which is an important character separating the Japanese *Monomorium* species as given in the key.

Another form, *M. intrudens pieli*, originally described as an independent species from Shanghai, China, also differs from *M. brachypterum* sp. nov. as mentioned below. According to the original description of the worker of *M. intrudens pieli* by Santschi (1925), the gaster is black except for the basal area of its venter (‘gaster noir sauf le dessous vers la base qui est jaunâtre’) (see also the image of the type: CASENT0913809 in Antweb, http://www.antweb.org, accessed 29 May 2025). However, in the new species, *M. brachypterum* n. sp., the dorsum of the gaster is more brownish and has yellowish areas of variable size. In the original description of *M. intudens pieli*, the antennal scape is said to nearly attain the posterior margin of the head (‘le scape attaint presque le bord poatérieur de la téte’). However, in the worker of the new species the length from the antennal base to the distal margin of the scape is around 82.2% of the distance from the antennal base to the posterior margin of the head (*N* = 5, SD = 0.028, range 0.769–0.866, Suppl. material 10: table S4), and naturally the scape does not reach the posterior margin of the head. Accordingly, *M. intrudens pieli* and the new species cannot be regarded as the same species. Overall, *M. brachypterum* can be distinguished from *M. intrudens* and *M. intrudens pieli* by worker morphology alone. However, our measurements of workers indicate partial overlap between the two species, and the morphological differences are more distinct in the queen caste. The undescribed queen of *M. intrudens pieli* will hopefully be examined in future studies.

Among social insects, it is difficult to find males larger than females under consistent selection for high fecundity (Stubblefield and Seger 1994; Boomsma et al. 2005). However, interestingly, Bolton (1987) noted that males in *Monomorium* range from slightly smaller to slightly larger than the queens. In both *M. intrudens* and *M. brachypterum*, the head width, the most commonly used body size index, overlaps between males and females, with males on average being slightly larger in the latter. Considering that male ants generally have smaller heads than queens (Shik et al. 2013), further research on the reduced sexual dimorphism in this group may provide a better understanding of the evolution of reproductive castes in social insects.

There are two main strategies for establishing a new nest in social insects: independent colony foundation and dependent colony foundation. The former is the basal and predominant strategy in which a winged queen disperses over long distances and establishes a new nest independently. The latter is a derived strategy in which a flightless queen disperses on foot to establish a new nest with the help of nestmate workers (Cronin et al. 2013). The queens of *M. brachypterum* had remarkably shortened wings compared to the queens of *M. intrudes*. Such short-winged queens are called “brachypterous queens” and they typically perform dependent colony founding (reviewed in Peeters 2012). This colony-level reproductive system has the advantage of skipping the initial stages of founding, when the colony is most vulnerable (Cronin et al. 2013). In fact, small (= immatured) colonies were not observed in *M. brachypterum*. These findings suggest *M. brachypterum* adopt a dependent colony foundation as the nest-level reproductive system. Although further morphological examination and molecular phylogenetic analysis of “*M. intrudens*” populations reported from outside Japan (South Korea, mainland of China, Taiwan and some introduced populations around Australasia, Antmaps, Janicki et al. 2016, accessed 13 June 2025) are needed, the more limited distribution of *M. brachypterum* compared to the wider distribution of *M. intrudens* may reflect differences in their dispersal abilities. Interestingly, very low variation was detected among the *COI* sequences of individuals from *M. brachypterum* populations. This lack of genetic variation could be associated with low dispersal ability or an unusual reproductive system, such as the female clonality reported in some congeneric species. Further research is warranted to explore this possibility.

Among the Japanese *Monomorium* species, *M. intrudens*, *M. chinense* and *M. hiten* produce normal winged queens while *M. floricola*, *M. triviale* and *M. brachypterum* produce wingless or brachypterous queens. The phylogenetic tree based on the complete mitochondrial genome suggests that the dependent colony founding queens occurred at least twice (once for *M. brachypterum*, once for *M. floricola* and *M. triviale*). It is consistent with the previous study, which reported that flightless queens are common (approximately 30% of the species in which the queen has been described) among species groups of *Monomorium* and may have evolved multiple times (Johnson and Overson 2017).

The genus *Monomorium* is also characterized by a diversity of reproductive systems at the individual level. Both *M. hiten* (Ito et al. 2021) and *M. triviale* (Idogawa et al. 2021b) have been reported as thelytokous parthenogenetic species. The phylogenetic analysis in the present study indicates that these two species are not sister species. Instead, a close relationship between thelytokous *M. triviale* and sexual *M. floricola* was suggested with high support values. This phylogenetic pattern implies that *M. hiten* and *M. triviale* acquired thelytoky independently. Together with the diversity of dispersal strategies, the present study emphasizes the potential of the genus *Monomorium* as a model system for the evolutionary pattern of colony- and individual-level reproductive systems in eusocial insects. However, molecular phylogenetic relationships of *Monomorium* species in East and Southeast Asia remain poorly understood due to limited sequence data available for these regions. We hope that this study will contribute to future efforts toward elucidating the global evolutionary history of *Monomorium*.

## Supplementary Material

XML Treatment for Monomorium brachypterum

## ﻿Additional information

### Use of AI

No use of AI was reported.

### Author ORCIDs

Naoto Idogawa https://orcid.org/0000-0003-4055-8204

Shigeto Dobata https://orcid.org/0000-0003-1586-6758

Yasukazu Okada https://orcid.org/0000-0003-4528-2980

Seiki Yamane https://orcid.org/0000-0002-6865-0865

### Data availability

All of the data that support the findings of this study are available in the main text or Supplementary Information.

## Supplementary materials

Supplementary material 1 List of collected nests Data type xls Explanation note A dataset on the social structure and sampling locations of *Monomorium brachypterum* and *M. intrudens*. https://binary.pensoft.net/file/1402311This dataset is made available under the Open Database License (http://opendatacommons.org/licenses/odbl/1.0/). The Open Database License (ODbL) is a license agreement intended to allow users to freely share, modify, and use this Dataset while maintaining this same freedom for others, provided that the original source and author(s) are credited.Naoto Idogawa, Shigeto Dobata, Yasukazu Okada, Seiki Yamane

Supplementary material 2 Results of measurements Data type xls Explanation note A dataset on the morphological measurements of *Monomorium brachypterum* and *M. intrudens*. https://binary.pensoft.net/file/1402312This dataset is made available under the Open Database License (http://opendatacommons.org/licenses/odbl/1.0/). The Open Database License (ODbL) is a license agreement intended to allow users to freely share, modify, and use this Dataset while maintaining this same freedom for others, provided that the original source and author(s) are credited.Naoto Idogawa, Shigeto Dobata, Yasukazu Okada, Seiki Yamane

Supplementary material 3 COI sequence data used in phylogenetic analysis Data type nexus https://binary.pensoft.net/file/1402313This dataset is made available under the Open Database License (http://opendatacommons.org/licenses/odbl/1.0/). The Open Database License (ODbL) is a license agreement intended to allow users to freely share, modify, and use this Dataset while maintaining this same freedom for others, provided that the original source and author(s) are credited.Naoto Idogawa, Shigeto Dobata, Yasukazu Okada, Seiki Yamane

Supplementary material 4 ML tree file based on COI sequences generated by IQ-TREE Data type contree https://binary.pensoft.net/file/1402314This dataset is made available under the Open Database License (http://opendatacommons.org/licenses/odbl/1.0/). The Open Database License (ODbL) is a license agreement intended to allow users to freely share, modify, and use this Dataset while maintaining this same freedom for others, provided that the original source and author(s) are credited.Naoto Idogawa, Shigeto Dobata, Yasukazu Okada, Seiki Yamane

Supplementary material 5 Bayesian tree file based on COI sequences generated by MrBayes Data type tre https://binary.pensoft.net/file/1402315This dataset is made available under the Open Database License (http://opendatacommons.org/licenses/odbl/1.0/). The Open Database License (ODbL) is a license agreement intended to allow users to freely share, modify, and use this Dataset while maintaining this same freedom for others, provided that the original source and author(s) are credited.Naoto Idogawa, Shigeto Dobata, Yasukazu Okada, Seiki Yamane

Supplementary material 6 13 PCGs sequence data used in phylogenetic analysis Data type nexus https://binary.pensoft.net/file/1402316This dataset is made available under the Open Database License (http://opendatacommons.org/licenses/odbl/1.0/). The Open Database License (ODbL) is a license agreement intended to allow users to freely share, modify, and use this Dataset while maintaining this same freedom for others, provided that the original source and author(s) are credited.Naoto Idogawa, Shigeto Dobata, Yasukazu Okada, Seiki Yamane

Supplementary material 7 ML tree file based on 13 PCGs sequences generated by IQ-TREE Data type contree https://binary.pensoft.net/file/1402317This dataset is made available under the Open Database License (http://opendatacommons.org/licenses/odbl/1.0/). The Open Database License (ODbL) is a license agreement intended to allow users to freely share, modify, and use this Dataset while maintaining this same freedom for others, provided that the original source and author(s) are credited.Naoto Idogawa, Shigeto Dobata, Yasukazu Okada, Seiki Yamane

Supplementary material 8 Bayesian tree file based on 13 PCGs sequences generated by MrBayes Data type tre https://binary.pensoft.net/file/1402318This dataset is made available under the Open Database License (http://opendatacommons.org/licenses/odbl/1.0/). The Open Database License (ODbL) is a license agreement intended to allow users to freely share, modify, and use this Dataset while maintaining this same freedom for others, provided that the original source and author(s) are credited.Naoto Idogawa, Shigeto Dobata, Yasukazu Okada, Seiki Yamane

Supplementary material 9 Summary of morphological comparisons between *Monomorium brachypterum* and *M. intrudens* workers across all populations Data type xls https://binary.pensoft.net/file/1402319This dataset is made available under the Open Database License (http://opendatacommons.org/licenses/odbl/1.0/). The Open Database License (ODbL) is a license agreement intended to allow users to freely share, modify, and use this Dataset while maintaining this same freedom for others, provided that the original source and author(s) are credited.Naoto Idogawa, Shigeto Dobata, Yasukazu Okada, Seiki Yamane

Supplementary material 10 A dataset on the measurements of scape length of *Monomorium brachypterum* workers Data type xls https://binary.pensoft.net/file/1402320This dataset is made available under the Open Database License (http://opendatacommons.org/licenses/odbl/1.0/). The Open Database License (ODbL) is a license agreement intended to allow users to freely share, modify, and use this Dataset while maintaining this same freedom for others, provided that the original source and author(s) are credited.Naoto Idogawa, Shigeto Dobata, Yasukazu Okada, Seiki Yamane
